# T-cell redirecting bispecific and trispecific antibodies in multiple myeloma beyond BCMA

**DOI:** 10.1097/CCO.0000000000000983

**Published:** 2023-07-24

**Authors:** Niels W.C.J. van de Donk, Chloe O’Neill, Maaike E.M. de Ruijter, Christie P.M. Verkleij, Sonja Zweegman

**Affiliations:** a Department of Hematology, Amsterdam UMC, location Vrije Universiteit Amsterdam; b Cancer Center Amsterdam, Cancer Biology and Immunology, Amsterdam, The Netherlands

**Keywords:** bispecific antibody, Fc receptor-homolog 5, G-protein-coupled receptor class 5 member D, multiple myeloma, trispecific antibody

## Abstract

**Purpose of review:**

B-cell maturation antigen (BCMA)-directed T-cell immunotherapies, such as chimeric antigen receptor T-cells (CAR T-cells) and bispecific antibodies (BsAbs) have markedly improved the survival of triple-class refractory multiple myeloma (MM). However, the majority of patients still develops disease progression, underlining the need for new agents for these patients.

**Recent findings:**

Novel T-cell redirecting BsAbs targeting alternative tumor-associated antigens have shown great promise in heavily pretreated MM, including patients previously exposed to BCMA-directed therapies. This includes the G-protein-coupled receptor class 5 member D (GPRC5D)-targeting BsAbs talquetamab and forimtamig, as well as the Fc receptor-homolog 5 (FcRH5)-targeting BsAb cevostamab. Toxicity associated with these BsAbs includes cytokine-release syndrome, cytopenias, and infections. In addition, GPRC5D-targeting BsAbs are associated with specific ‘on target/off tumor’ toxicities including rash, nail disorders, and dysgeusia. Trispecifc antibodies targeting two different MM-associated antigens to prevent antigen escape are in early clinical development, as well as trispecific antibodies (TsAbs) that provide an additional co-stimulatory signal to T-cells to prevent their exhaustion.

**Summary:**

Various T-cell redirecting BsAbs are in advanced stages of clinical development with promising activity and a manageable toxicity profile. Ongoing studies are evaluating combination strategies, fixed-duration treatment, and use of BsAbs in earlier lines of therapy. TsAbs hold great promise for the future.

## INTRODUCTION

The survival of multiple myeloma (MM) patients has markedly improved over the last two decades because of introduction of several novel classes of anti-MM drugs including immunomodulatory drugs (IMiDs; thalidomide, lenalidomide and pomalidomide), proteasome inhibitors (bortezomib, ixazomib, and carfilzomib), and naked antibodies (anti-CD38 antibodies [daratumumab and isatuximab] and SLAM family member 7 (SLAMF7)-targeting antibodies [elotuzumab]) [1]. These drugs are most often used in synergistic combinations to prevent outgrowth of resistant clones.

However, patients who develop disease that is refractory to these three different drug classes (triple-class refractory disease) have a very poor survival, indicating that there is an unmet need for new agents with different mode of action [2–4]. During the past few years various clinical studies were initiated to evaluate novel agents in patients with triple-class exposed or triple-class refractory MM. This has led to the recent approval of a number of new agents in this setting including the nuclear export inhibitor selinexor [5] and the B-cell maturation antigen (BCMA)-targeting antibody-drug conjugate (ADC) belantamab mafodotin [6]. However, based on negative results from the confirmatory phase 3 DREAMM-3 study (no significant difference between belantamab mafodotin and pomalidomide-dexamethasone in patients who were treated with at least 4 prior therapies, including an anti-CD38 antibody, a proteasome inhibitor, and an IMiD) belantamab mafodotin was recently withdrawn from the US market. Belantamab mafodotin is still approved in Europe by EMA as monotherapy for patients with at least four prior lines of therapy and triple-class refractory disease [6]. Belantamab mafodotin-containing combination studies are ongoing [7].

Most promising results in the setting of heavily pretreated MM were obtained with novel T-cell immunotherapies, both CAR T-cell therapies and T-cell redirecting bispecific antibodies (BsAbs) [8–11]. The majority of these novel T-cell immunotherapies are targeting the MM-associated antigen BCMA. This cell surface protein is an attractive target for highly active T-cell immunotherapies because of its selective expression on normal and malignant plasma cells, as well as on mature B cells [12]. The selective expression reduces the potential for severe ‘on target/off tumor’ toxicity. However, a disadvantage is the depletion of normal B cells and normal plasma cells, which frequently results in severe hypogammaglobulinemia and increased risk of infections [13^▪▪^,^14^,^15^]. The approved CAR T-cell products ide-cel [16] and cilta-cel [17] target BCMA, and also the T-cell redirecting BsAb teclistamab targets BCMA [13^▪▪^]. Teclistamab was the first BsAb that received regulatory approval based on high efficacy (overall response rate (ORR): 63.0%; ≥complete response (CR): 45.5%; median progression-free survival (PFS): 11.3 months; median response duration: 21.6 months) with a manageable toxicity profile in patients with advanced MM (triple-class refractory: 77.6%) [10,13^▪▪^]. Elranatamab (BCMAxCD3 BsAb) has also shown substantial activity in heavily pretreated MM (ORR: 61.0%; ≥CR: 35.0%; PFS at 15 months: 50.9%) [14,18]. Several other BCMA-targeting BsAbs are in clinical development with comparable activity and safety profile, but with some differences in terms of mode of administration (intravenous (IV) or subcutaneous (SC) administration) or frequency of administration [14,19–22]. Although BCMA-targeting T-cell therapies are highly active, the majority of patients eventually develops disease progression.

Resistance mechanisms to T-cell immunotherapies include tumor-related features, T-cell characteristics, as well as features from the immunosuppressive tumor microenvironment [23]. BCMA reduction is observed after CAR T-cell therapy [24], but BCMA loss seems to be a rare event (2–3%) [16,25–27]. Loss of BCMA expression due to BCMA gene deletions or mutations seems to be a more common cause of acquired resistance to BCMA-targeting BsAbs [28,29], which may be related to the long-term treatment with these agents, as opposed to the single infusion of chimeric antigen receptor T-cells (CAR T-cells). Sequencing of BCMA therapies can be effective, but it has also been shown that BCMA CAR T-cell therapy is less effective directly following other non-cellular BCMA-directed therapies (both BCMA BsAbs and BCMA ADCs) [30–32]. In addition, although BCMA BsAbs are effective after prior BCMA-directed therapy (CAR T-cell therapy or ADC), activity is slightly reduced in patients with prior BCMA-targeted therapy. The overall response rate with teclistamab in patients with prior BCMA CAR T-cell therapy or BCMA ADC was 55.2% and 53.3%, respectively [33]. Comparable results were observed with elranatamab in patients with prior BCMA-directed therapy (overall response rate after prior ADC: 42.4%; overall response rate after prior BCMA CAR T-cell therapy: 52.8%) [34]. These findings may be explained in part by BCMA antigen modulation, but effects of prior BCMA targeted therapies on T-cell fitness may also play a role [35]. An alternative therapeutic regimen that has the potential to improve T-cell function (e.g, CD38 antibody-based regimen or IMiD/Cereblon E3 ligase modulator (CELMoD)-containing regimen) could be valuable as a bridge between two different T-cell immunotherapies.

Given the increased use of BCMA-targeted therapies, and the potential to develop BCMA loss or mutations during BCMA-targeted therapy, there is a strong need for novel agents targeting alternative MM-associated antigens. Interestingly, a single center study recently showed that after treatment with a BsAb and disease relapse, MM patients could be effectively salvaged with sequential T-cell redirection therapy (either BsAb or CAR T-cell therapy) with superior results compared to other types of anti-MM therapy [36]. In this study most patients switched target either from G-protein-coupled receptor class 5 member D (GPRC5D) to BCMA, or BCMA to GPRC5D [36].

In this review, we will discuss the efficacy and safety profile of novel T-cell redirecting antibodies targeting alternative tumor-associated antigens that are currently under clinical evaluation. Most advanced in terms of clinical testing are BsAbs targeting GPRC5D and Fc receptor-homolog 5 (FcRH5). 

**Box 1:**
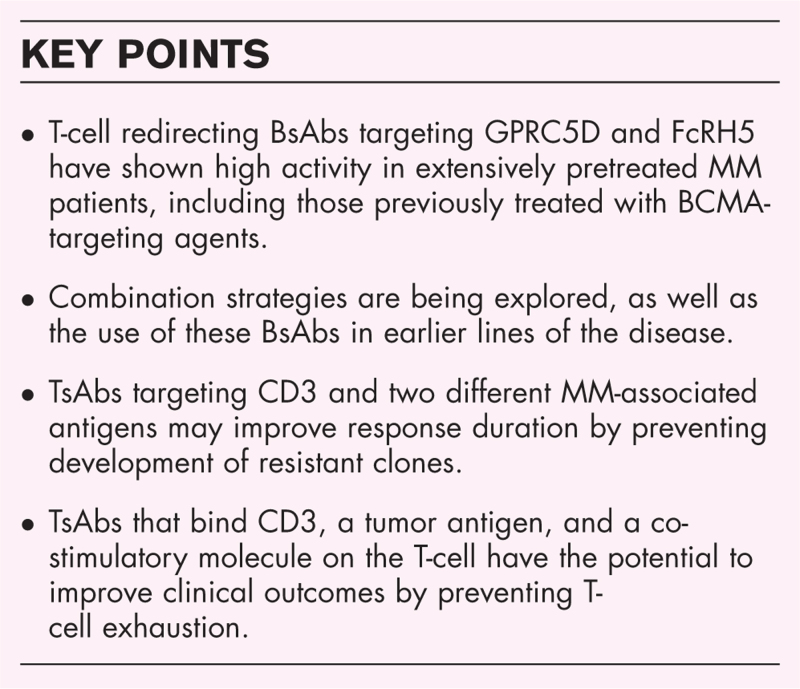
no caption available

## TARGETING G-PROTEIN-COUPLED RECEPTOR CLASS 5 MEMBER D

**FIGURE 1:**
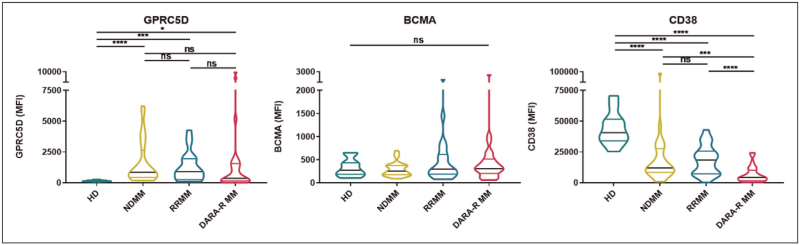
Cell surface expression of GPRC5D, BCMA and CD38. Protein levels on the surface of plasma cells from healthy donors or MM patients were assessed by flow cytometry. Expression of GPRC5D is lower on normal plasma cells compared to malignant plasm cells, while there is no difference for BCMA and expression of CD38 is higher on normal plasma cells. The violin plots visualize the distribution of target expression levels with the black line representing the median and the colored lines represent the first and third quartiles. Adapted from Verkleij *et al.*[37] with permission. ∗*P* < .05; ∗∗∗*P* < .001; ∗∗∗∗*P* < .0001. DARA-R MM, daratumumab-refractory MM; HD, healthy donor plasma cells; NDMM, newly diagnosed MM; ns, not significant; RRMM, daratumumab naïve relapsed/refractory MM.

GPRC5D is a transmembrane receptor protein, which is highly expressed on the surface of MM cells, but up till now its function remains unknown. The expression of GPRC5D is significantly lower on normal plasma cells, compared to malignant plasma cells (Fig. 1) [37]. In contrast, expression of other MM-associated antigens is comparable (BCMA [37,38] and FcRH5 [39]) or lower (CD38 [40]) on malignant plasma cells versus normal plasma cells (Fig. 1). Such differences may explain differences in the frequency of infections between GPRC5D-targeting BsAbs and those targeting BCMA. GPRC5D is also expressed on nonhematopoietic tissues including hair follicles, filiform papillae of the tongue, and epithelial cells of the eccrine sweat glands in the skin [41–43]. Expression of GPRC5D has also been demonstrated in the nail bed of mice, but this has yet to be confirmed in humans [44].

### Talquetamab

**FIGURE 2:**
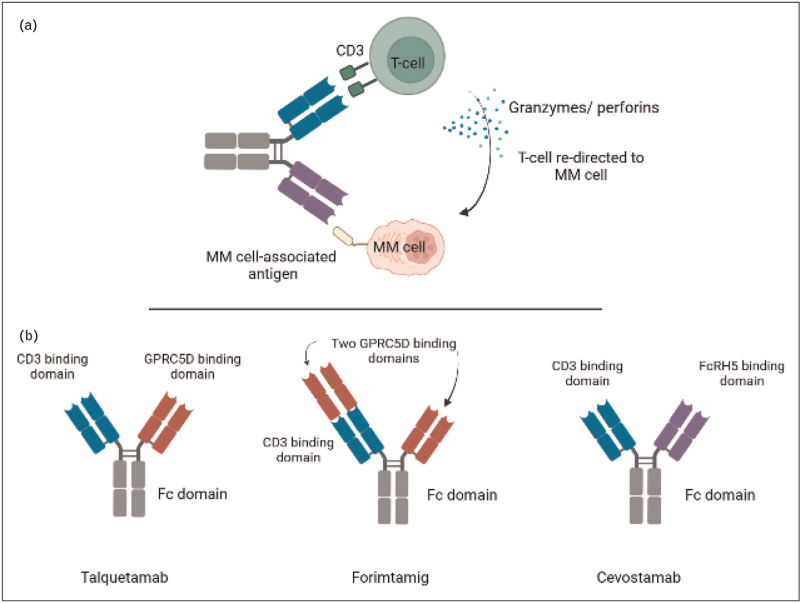
BsAbs targeting GPRC5D and FcRH5 in advanced stages of clinical development for the treatment of MM. (A) A T-cell redirecting bispecific antibody binds simultaneously to CD3 on the T-cell and a target antigen on the MM cell. This results in the formation of an immune synapse and then T-cell activation/degranulation with release of granzymes and perforins, and subsequent MM cell death. (B) Molecular configuration of talquetamab, forimtamig, and cevostamab. Picture is created with BioRender.com.

Talquetamab is the first-in-class GPRC5D-targeting BsAb. By simultaneously binding to CD3 on T-cells and GPRC5D on MM cells, T-cells are redirected to the tumor cells resulting in the formation of an immune synapse [37,42] (Fig. 2). This is followed by T-cell activation and degranulation with release of granzymes and perforins, leading to tumor cell death.

In the dose-escalation part of the MonumenTAL-1 study two different recommended phase 2 dose levels (RP2D) were defined: 0.4 mg/kg with weekly SC administration (2 step-up doses) and 0.8 mg/kg with SC administration every 2 weeks (3 step-up doses) [45^▪▪^].

Patients also received premedication with a glucocorticoid, antihistamine, and acetaminophen prior to all step-up doses and initial full dose. Additional patients (143 with 0.4 mg/kg and 145 with 0.8 mg/kg) were treated with talquetamab dosed with these RP2Ds (median of 5 prior lines of therapy; triple-class refractory: 69.0–74.1%; Table 1) [46,47]. The overall response rate was 74.1% in the 0.4 mg/kg cohort (≥very good partial response (VGPR): 59.4%) and 71.7% in the 0.8 mg/kg cohort (≥VGPR: 60.7%). Response was lower in patients with plasmacytomas, which may be related to aggressive underlying tumor biology and a more immune suppressive microenvironment in such tumors [46,47]. The median PFS was 7.5 months in the 0.4 mg/kg cohort and 14.2 months in the 0.8 mg/kg cohort, with median response duration of 9.5 months and not yet reached, respectively [46–48]. A total of 51 patients were previously exposed to other T-cell redirection therapies (CAR T-cell therapy: 70.6%; BsAb: 35.3%; both CAR T-cell therapy and BsAb: 6.0%) [46,47]. Although these patients had already been exposed to T-cell immunotherapy, the overall response rate (64.7%) and response duration with talquetamab (median of 11.9 months) are promising [46,47]. The overall response rate was 75.0% in patients with prior CAR T-cell therapy and 44.4% in patients with prior BsAb treatment [47].

**Table 1:** Key characteristics of T-cell redirecting BsAbs targeting GPRC5D or FcRH5

BsAb	Design	n	Median follow-up	Triple-class refractory (%)	Prior lines (median)	Partial response rate/complete response rate (%)	Median PFS	CRS (all grade/grade≥3) (%)	ICANS (all grade) (%)	Infections (all grade/ grade≥3) (%)
Talquetamab [45^▪▪^,^46^,^47^,^48^]	SC, 2-3 step-up doses, Q1W or Q2W	Two RP2Ds: 143 patients treated at the 400 μg/kg QW dosing schedule, and 145 patients treated at the 800 μg/kg Q2W dosing schedule	400 μg/kg: 18.8 months 800 μg/kg: 12.7 months	400 μg/kg: 74.1 800 μg/kg: 69.0	400 μg/kg: 5 800 μg/kg: 5	400 μg/kg: 74.1/33.6 800 μg/kg: 71.7/38.7	400 μg/kg: 7.5 months 800 μg/kg: 14.2 months	400 μg/kg: 79.0/2.1 800 μg/kg: 74.5/0.7	400 μg/kg: 10.7 800 μg/kg: 11.0	400 μg/kg: 58.7/19.6 800 μg/kg: 66.2/14.5
Forimtamig (RG6234) [49^▪▪^]	IV or SC, 2 step-up doses, Q2W for 1 year	IV arm: 51 SC arm: 57	IV arm: 11.6 months SC arm: 8.0 months	IV arm: 62.0 SC arm: 71.9	IV arm: 5 SC arm: 4	IV arm: 71.4/34.7 SC arm: 63.6/25.5	NR	IV arm: 82.4/2.0 SC arm: 78.9/1.8	IV arm: 9.8 SC arm: 12.3	IV arm: 60.8/21.5 SC arm: 45.6/26.4
Cevostamab [50^▪▪^]	IV, 1-2 step-up doses, Q3W	161	Single step-up cohorts (responders): 14.3 months Double step-up cohorts (responders): 6.5 months	84.5	6	132-198 mg: 56.7/8.4	NR	80.7/1.2	14.3	Approximately 45% (20%)

ICANS, immune effector cell-associated neurotoxicity syndrome; IV, intravenous; NR, not reported; RP2D, recommended phase 2 dose; SC, subcutaneous.

**FIGURE 3:**
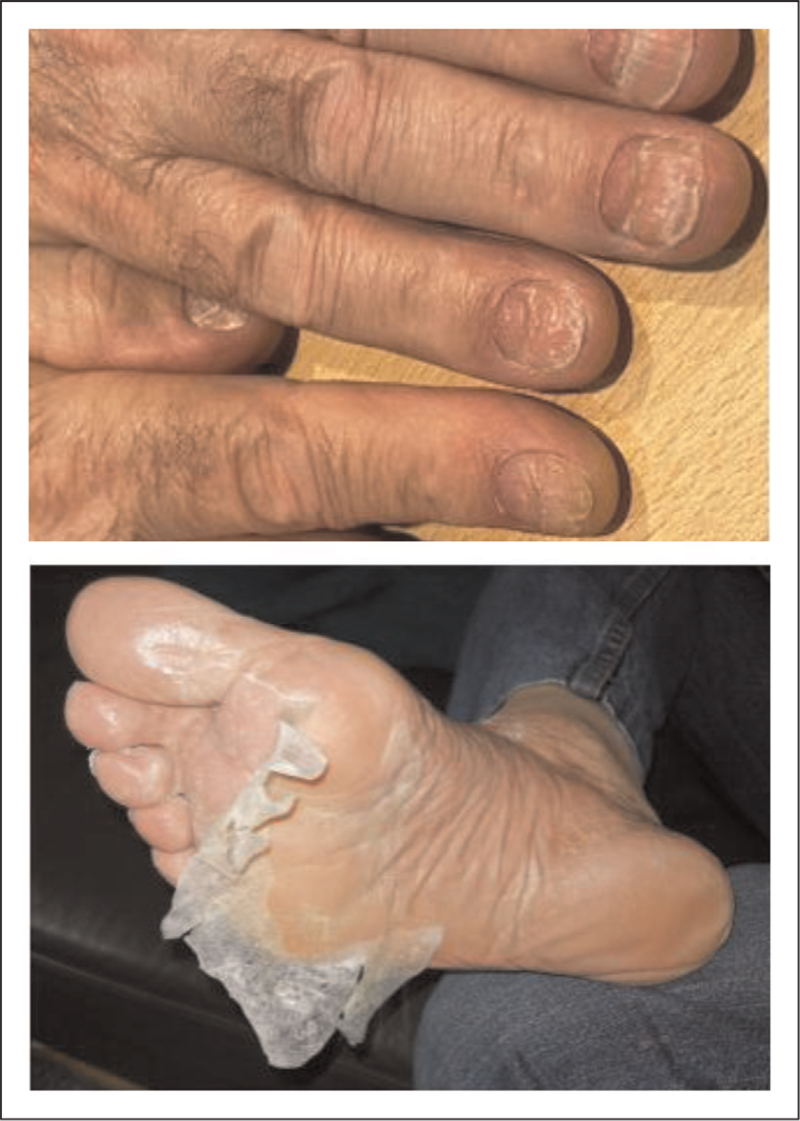
On target/off tumor toxicities associated with GPRC5D-targeting BsAbs. (A) Nail changes. (B) Palmar desquamation.

As expected, the talquetamab-mediated T-cell activation led to development of cytokine release syndrome (CRS) in the majority of patients (74.5–79.0%), but CRS was typically grade 1 or 2, with grade 3 CRS in only 0.7–2.1% of patients [46,47]. Most CRS events occurred during step-up dosing or following the first full dose. CRS could be effectively managed with supportive care such as tocilizumab. Immune effector cell-associated neurotoxicity syndrome was an uncommon side effect (10.7–11.0%; mostly grade 1 or 2), often in the setting of CRS. Other common adverse events included hematologic adverse events, which were most frequent during the first 1 to 2 treatment cycles [46,47]. Infections occurred in 58.7% (≥grade 3: 19.6%) and 66.2% (≥grade 3: 14.5%) in the 0.4 and 0.8 mg/kg cohorts, respectively [46,47]. Although there are substantial differences in follow-up among the different studies, the frequency of infections with talquetamab seems lower than what is observed with BCMA-targeting BsAbs (Table 2) [10,14,47,49^▪▪^,^50^^▪▪^,^51^]. This may be explained by sparing of normal plasma cells and normal B cells with GPRC5D-targeting BsAbs, because of low GPRC5D expression on normal plasma cells and no expression on normal B cells [52], while BCMA-targeting BsAbs deplete both normal plasma cells and normal B cells [15]. Indeed, there is no decrease in immunoglobulin G (IgG) levels during talquetamab treatment [52], while treatment with BCMA-targeting BsAbs is associated with a marked decrease in polyclonal IgG levels [15]. IVIG supplementation therapy was also more frequently administered to patients treated with BCMA-targeting BsAbs, compared to GPRC5D-targeting BsAbs [10,46]. Nonetheless, opportunistic infections have been observed during talquetamab treatment including cytomegalovirus infection, fungal sepsis, adenovirus infection, and herpes infections) [46,47,52]. T-cell exhaustion because of continuous T-cell activation may contribute to development of opportunistic infections, but also the high cumulative exposure to a variety of immunosuppressive anti-MM agents during the patient's disease course may have contributed to these rare infections [53]. The targeting of GPRC5D on the surface of cells that produce keratin is most likely responsible for the development of the GPRC5D-specific ‘on target/off tumor’ adverse events, including skin-related adverse events (rash, as well as palmar/plantar desquamation [Fig. 3]), nail-related adverse events, and dysgeusia [46,47]. Skin toxicities can be managed with supportive care (emollients, triamcinolone cream, and oral steroids for more severe cases) [54]. Dysgeusia is typically managed by supportive care (e.g., saliva substitute spays and rinses) combined with dose reduction or less frequent administration [54]. In our practice dysgeusia can be fully reversible with such dose modifications, but this may take several months. Support by a dietician can also be helpful in case of dysgeusia and/or weight loss [54].

**Table 2:** Frequency of infections in patients treated with BCMA and non-BCMA-targeting BsAbs

Bispecific antibody	Target	Follow-up (median)	Any grade infections	≥Grade 3 infections
Teclistamab [10]	BCMA	23 months	80%	55%
Elranatamab [14,18]	BCMA	15 months	70%	46%
Talquetamab (0.4 mg/kg QW; 0.8 mg/kg Q2W dose) [47]	GPRC5D	19/13 months	59/66%	20/15%
Forimtamig (IV/SC) [49^▪▪^]	GPRC5D	11.6/8.0 months	61/46%	22/26%
Cevostamab [50^▪▪^]	FcRH5	6.1 months	43%	19%

Of note, there are substantial differences in follow-up among studies, which also explains part of the differences in the frequency of infections. BCMA, B-cell maturation antigen; BsAbs, bispecific antibodies; IV, intravenous; SC, subcutaneous.

A cerebellar disorder occurred in two patients treated with GPRC5D-targeting CAR T-cells (MCARH109) at the highest dose-level [55]. Symptoms included dizziness and unsteady gait [55]. This novel toxicity is probably caused by expression of GPRC5D in the inferior olivary nucleus [55], and therefore CAR T-cell-mediated damage in that region of the brain. To the best of our knowledge, so far, no cerebellar symptoms have been observed with GPRC5D-targeting BsAbs.

The TRIMM-2 study is evaluating various talquetamab-based combinations. Preliminary data from the combination of talquetamab plus daratumumab showed a promising efficacy profile with toxicity in line with what is observed with both agents alone [56,57]. The preclinical rationale for this combination regimen is the immune-modulatory effect of daratumumab including elimination of regulatory T-cells as well as the induction of T-cell expansion and improvement in T-cell cytolytic activity [37,38,58^▪▪^,^59^,^60^,^61^]. Based on these results a phase 3 study is currently evaluating talquetamab in combination with daratumumab or in combination with daratumumab and pomalidomide versus daratumumab-pomalidomide-dexamethasone (MonumenTAL-3 study; NCT05455320).

Talquetamab has also been evaluated in a combination with teclistamab in 93 heavily pretreated MM patients (median 4 prior lines of therapy; 79.6% triple-class refractory). Across all dose levels, at least partial response (PR) was achieved by 86.6% of patients, including ≥CR in 40.2% [62]. This translated into a median PFS of 20.9 months [62]. Although number of patients is relatively small (*n* = 35), this dual-targeting approach was especially effective in patients with soft tissue plasmacytomas. These high-risk patients experience a relatively low response rate with BCMA- or GPRC5D-targeting BsAbs, but they obtained pronounced benefit from the combination of teclistamab and talquetamab (ORR: 71.4%; ≥CR: 21.4; median PFS: 6.1 months) [62].

An ongoing study is also evaluating talquetamab in combination with a programmed cell death protein-1 (PD-1) inhibitor (TRIMM-3). This combination strategy is based on promising preclinical data showing that the efficacy of T-cell redirecting antibodies can be enhanced by concomitant PD-1/programmed death-ligand 1 (PD-L1) blockade [63].

### Forimtamig

Forimtamig is another CD3xGPRC5D BsAb, with two binding domains for GPRC5D, which confers high affinity binding to MM cells (Fig. 2). In preclinical studies forimtamig has demonstrated substantial anti-MM activity [64]. Forimtamig is currently being evaluated in a dose-escalation phase 1 study with both SC and IV administration (Table 1) [49^▪▪^]. Patients also received corticosteroid premedication during cycle 1 to mitigate CRS. In heavily pretreated patients (triple-class refractory: 62.0–71.9%) forimtamig showed potent anti-MM activity across all tested target doses with an overall response rate of 71.4% (≥VGPR: 59.2%) in the IV arm and 63.6% (≥VGPR: 52.8%) in the SC arm [49^▪▪^]. Median duration of response was 10.8–12.5 months [49^▪▪^]. Overall response rate in the patients with prior BCMA-directed therapy (CAR T-cell therapy, BsAb, or ADC) was 52.4% [49^▪▪^]. The toxicity profile was comparable to that of talquetamab, including development of dysgeusia, dry mouth, and skin rash [49^▪▪^]. The SC administration induced delayed and lower cytokine secretion compared with the IV infusion, which explains that the median time to CRS onset is shorter for IV dosing (5 h), compared to 24 h for SC dosing [49^▪▪^].

Preclinical results have shown that forimtamig-mediated MM cell killing can be enhanced by daratumumab and pomalidomide [64]. This forms the rationale for combination strategies with forimtamig.

## TARGETING Fc RECEPTOR-HOMOLOG 5

FcRH5 is exclusively expressed in the B-cell lineage with higher expression on MM cells compared to normal B-cells [39]. Cevostamab targets CD3 and FcRH5 and thereby is capable of inducing T-cell redirected killing of MM cells (Fig. 2) [39]. Promising activity was observed in the phase 1 dose-finding trial (Table 1). which enrolled heavily pretreated patients (median of 6 prior lines; triple-class refractory: 84.5%) [50^▪▪^]. Cevostamab was administered IV every 3 weeks for a maximum of 17 cycles (approximately 1 year) with premedication to mitigate CRS (acetaminophen, antihistamine, and corticosteroid). At the higher dose levels, the overall response rate was 56.7% (≥VGPR: 33.3%) [50^▪▪^]. Median response duration was 11.5 months in the single step-up cohorts [50^▪▪^]. A subgroup analysis of 18 patients who stopped treatment after 17 cycles while in remission, showed that most responders (78%) remained in response (median follow-up 9.6 months) [65]. Also, patients who discontinued cevostamab treatment because of adverse events after a median of time on treatment of 6 months, were able to maintain their response (median duration of response after treatment discontinuation: 9.2 months) [65]. These data indicate that fixed-duration treatment can be effective. A treatment-free period may also allow for reversal of T-cell exhaustion and potentially reduces risk of infections. Based on these and other data, fixed-duration treatment should also be explored with other BsAbs.

Hematologic toxicity, infections and CRS were the most common adverse events with cevostamab [50^▪▪^]. CRS occurred mostly during the first treatment cycle with rare occurrence of grade 3 events (1.2%) [50^▪▪^]. Double step-up dosing was associated with an improve CRS profile compared to one step-up dose [50^▪▪^]. Interestingly, prophylactic tocilizumab (tocilizumab administered 1 h before the first cevostamab dose) further reduced the CRS incidence from 90.9% (grade 2: 34.1%; grade 3: 2.3%) to 38.7% (grade 2: 16.1%; grade 3: 3.2%) [66^▪^]. Prophylactic tocilizumab use increased the frequency of neutropenia, but this was reversible and manageable with growth factor support where needed [66^▪^]. There was no negative impact on response with prophylactic use of tocilizumab.

## OTHER TARGETS FOR T-CELL REDIRECTING BISPECIFIC ANTIBODIES

Several other targets are being explored to generate novel BsAbs or trispecific antibodies (TsAbs; see next section). One of these alternative targets is CD38, which is highly expressed on MM cells and already used as target for naked antibodies (daratumumab and isatuximab) [67]. It functions as a receptor for CD31 and also has ectoenzymatic activities (nicotinamide adenine dinucleotide glycohydrase (NADase)) [68]. Next to expression in the hematopoietic system, it is also expressed at low levels in other tissues of nonhematopoietic origin including prostatic epithelial cells, pancreatic islet cells, as well as in the perikarya and dendrites of some neurons. Other CD38-positive cells include airway-striated muscle cells, renal tubules, retinal gangliar cells, and corneal cells [69].

Clinical development of the CD38xCD3 BsAb AMG 424 was halted (business decision) [70]. However, other CD38-targeting BsAb are in early stages of clinical evaluation. This includes ISB 1342, which binds with high affinity to CD38 [71]. Importantly the epitope is different from that of daratumumab or isatuximab [71]. Preclinical experiments showed superior potency of ISB 1342, compared to daratumumab, against tumor cells with both high and low CD38 expression [71]. This formed the rationale for an ongoing phase 1 dose-escalation study. Preliminary results provide evidence of T-cell activation following ISB 1342 administration and a manageable toxicity profile [71].

Another BsAb targeting CD38 is IGM-2644. This agent is an IgM BsAb that can also mediate complement-dependent cytotoxicity [72]. It has ten binding sites for human CD38, and a single anti-CD3 binding domain [72]. Based on promising preclinical data, a phase 1 clinical trial is planned to study the activity and safety of IGM-2644 in advanced MM [72].

A potential novel target for targeted therapy in MM is immunoglobulin-like transcript 3 (ILT3), which is an immunoreceptor tyrosine-based inhibition motif-containing receptor, with high expression on MM cells and low or absent expression in normal tissues [73]. A CD3xILT3 BsAb showed promising anti-MM activity in preclinical experiments [73].

CD1d is expressed on tumor cells in the majority of patients with MM or chronic lymphocytic leukemia and in a subset of patients with acute myeloid leukemia [74]. LAVA-051 is a bispecific single domain antibody that directly engages CD1d and the Vδ2-T cell receptor chain of Vγ9Vδ2-T cells and additionally stabilizes the interaction between CD1d and type 1 natural killer T cells to mediate potent killing of CD1d-expressing tumor cells [74]. It is currently evaluated in a phase 1 dose-escalation study in heavily pretreated MM.

## FUTURE DEVELOPMENTS

Heterogeneity in target expression within the tumor may lead to the outgrowth of resistant clones [23]. In addition, acquired antigen loss or antigen downregulation during treatment may also lead to therapy failure [28,29]. T-cell redirecting TsAbs that simultaneously target two different MM-associated antigens have the potential to prevent or delay the outgrowth of resistant clones due to heterogeneity or changes in antigen expression.

One of the TsAbs in clinical development is JNJ-79635322. This agent simultaneously targets CD3 on T-cells and BCMA and GPRC5D on tumor cells. A more targeted delivery of this agent to the tumor because of binding to two MM-associated antigens may also lead to lower ‘on target/off tumor’ toxicity, and this will be carefully evaluated in the ongoing clinical trial.

Another TsAb, SAR442257, binds next to CD3, also to CD38 and CD28 [75]. CD28 is expressed on MM cells in approximately one third of newly diagnosed MM patients [76], and in these tumors the TsAb will probably have increased binding strength. More important, CD28 is also a costimulatory molecule expressed on T-cells. Interaction of the TsAb with CD28 provides a costimulatory signal to the T-cell and thereby prevents T-cell exhaustion. Altogether these properties translated into potent anti-MM activity in preclinical MM models. An ongoing phase 1 study is evaluating safety and efficacy of SAR442257 in patients with advanced MM.

Other TsAbs evaluated in MM include ISB 2001 (targeting CD3, CD38 and BCMA), which showed promising activity in preclinical MM models [77]. A phase 1 dose-escalation study is planned with this drug in heavily pretreated MM.

The combination of a BsAb with other novel anti-MM agents may also improve depth and duration of response. Several combination strategies are currently being evaluated in both preclinical and/or clinical settings. Cereblon E3 ligase modulatory drugs (CELMoDs) (iberdomide and mezigdomide) bind with higher affinity than IMiDs to the target Cereblon, which translates into increased direct anti-MM activity as well as more potent immune stimulatory effects [78^▪▪^,^79^]. CELMoDs are therefore promising partner drugs for BsAbs in MM, and possibly also in other malignancies. It has recently been shown that both iberdomide and mezigdomide have the ability to enhance the antitumor effect of BsAbs [80,81]. In *in vitro* experiments CELMoDs also have the ability to reduce BsAb-mediated pro-inflammatory cytokine secretion by myeloid cells (e.g. interleukin-6 and IL-1β), and therefore these agents also hold promise to mitigate CRS [82].

Preclinical experiments have shown that the efficacy of BsAbs can also be improved by blocking inhibitory receptors on T-cells. for example blockade of the PD-1/PD-L1 signaling pathway improved the antitumor activity of BsAbs [63]. Based on the data, several studies are now evaluating the combination of a BsAb and a checkpoint inhibitor in patients with heavily pretreated MM.

BsAbs are also moving to earlier lines of therapy where T-cell fitness is better than what is observed in patients with heavily pretreated disease [83]. Up till now only BCMA-targeting BsAbs are being evaluated in newly diagnosed disease, but studies with BsAbs targeting other antigens in patients with less pretreatment are ongoing, such as the MonumenTAL-3 study, which evaluates the efficacy of talquetamab-based combination therapy in patients with at least one prior line of therapy.

## CONCLUSION

T-cell redirecting BsAbs targeting GPRC5D and FcRH5 have demonstrated high activity with a manageable toxicity profile in heavily pretreated MM, including those previously exposed to BCMA agents. Novel combination strategies and earlier use of these agents may lead to further improvements in depth and duration of response. In addition, TsAbs targeting two different tumor antigens hold great promise.

## Acknowledgements

*None.*

### Financial support and sponsorship

*None.*

### Conflicts of interest

*N.W.C.J.v.d.D. has received research support from Janssen Pharmaceuticals, AMGEN, Celgene, Novartis, Cellectis and BMS, and serves in advisory boards for Janssen Pharmaceuticals, AMGEN, Celgene, BMS, Takeda, Roche, Novartis, Bayer, Adaptive, and Servier, all paid to institution; S.Z. has received research funding from Celgene, Takeda, Janssen, and serves in advisory boards for Janssen, Takeda, BMS, Oncopeptides and Sanofi, all paid to institution; all other authors declared no conflicts of interest.*

## REFERENCES AND RECOMMENDED READING

Papers of particular interest, published within the annual period of review, have been highlighted as:

▪ of special interest
▪▪ of outstanding interest
